# RSF Governs Silent Chromatin Formation via Histone H2Av Replacement

**DOI:** 10.1371/journal.pgen.1000011

**Published:** 2008-02-29

**Authors:** Kazuma Hanai, Hirofumi Furuhashi, Takashi Yamamoto, Koji Akasaka, Susumu Hirose

**Affiliations:** 1 Department of Developmental Genetics, National Institute of Genetics, Shizuoka-ken, Japan; 2 Department of Mathematical and Life Science, Graduate School of Science, Hiroshima University, Higashi-Hiroshima, Japan; 3 Misaki Marine Biological Station, Graduate School of Science, The University of Tokyo, Miura, Kanagawa, Japan; European Molecular Biology Laboratory, Germany

## Abstract

Human remodeling and spacing factor (RSF) consists of a heterodimer of Rsf-1 and hSNF2H, a counterpart of *Drosophila* ISWI. RSF possesses not only chromatin remodeling activity but also chromatin assembly activity in vitro. While no other single factor can execute the same activities as RSF, the biological significance of RSF remained unknown. To investigate the in vivo function of RSF, we generated a mutant allele of *Drosophila Rsf-1* (*dRsf-1*). The *dRsf-1* mutant behaved as a dominant suppressor of position effect variegation. In *dRsf-1* mutant, the levels of histone H3K9 dimethylation and histone H2A variant H2Av were significantly reduced in an euchromatic region juxtaposed with heterochromatin. Furthermore, using both genetic and biochemical approaches, we demonstrate that dRsf-1 interacts with H2Av and the H2Av-exchanging machinery Tip60 complex. These results suggest that RSF contributes to histone H2Av replacement in the pathway of silent chromatin formation.

## Author Summary

As DNA is packaged into chromatin in the nucleus, every DNA transaction requires alteration of the chromatin structure. RSF, a heterodimer of Rsf-1 and ISWI/SNF2H, is a unique chromatin remodeling factor that can assemble regularly spaced nucleosome arrays without the aid of histone chaperons, but its biological function is not clear. Using *Drosophila melanogaster* as a model organism, we investigated the in vivo role of RSF in gene expression. The loss of RSF function reduces the levels of histone variant H2Av and histone H3-K9 methylation, and suppresses silencing of transcription in an euchromatic region neighboring the centromeric heterochromatin. We also observed that Rsf-1 interacts with histone H2Av and the H2Av-exchanging machinery Tip60 complex. Based on these findings, we propose that RSF plays a role in silent chromatin formation by promoting histone H2Av replacement.

## Introduction

In the eukaryotic nucleus, DNA is packaged as a chemically stable nucleoprotein called chromatin. The fundamental unit of chromatin is a nucleosome, which is made up of 146 bp of DNA wrapped around an octamer of histone proteins H2A, H2B, H3 and H4 [1]. Therefore, every nuclear process that requires access to DNA proceeds in the context of chromatin. This makes possible to regulate the process through the chromatin structure. To stabilize or overcome the inhibitory effect of chromatin, some mechanisms that alter the chromatin structure are necessary. One of the mechanisms for modulation of the chromatin structure is modification of histones by acetylation, phosphorylation, methylation, ubiquitination, or ADP-ribosylation [2],[3]. Recently, histone variants have been also shown to be important for modulating chromatin status [4]. The majority of variants that have been reported to date correspond to two types of histones, H2A and H3. Many of the H2A variants are involved in the formation of higher-order chromatin structure [5]. H2A.Z, one of the best-studied H2A variants, is essential for establishing proper chromatin structure in many organisms [6]. For example, mammalian H2A.Z contributes to unique structure of centromere [7] and is essential to maintain the genome integrity [8]. In addition to the function of H2A.Z in constitutive heterochromatin, H2A.Z also has a role for formation of facultative heterochromatin [9],[10]. *Drosophila melanogaster* has a single H2A.Z variant, H2Av that comprises 5–10% of cellular H2A protein [11]. As mammalian H2A.Z, H2Av nonrandomly localizes to heterochromatin regions and many euchromatin regions on polytene chromosomes [12], A recent study has demonstrated that chromatin in *Drosophila* H2Av mutants exhibits reduced H3 lysine 9 (H3K9) methylation and reduced HP1 binding and Pc binding [13], suggesting an essential role of H2Av for silent chromatin formation in *Drosophila*.

Another important modulator of chromatin regulation is ATP-dependent chromatin remodeling, which alters the chromatin structure or positioning of nucleosomes. ISWI is one of the highly conserved and well-characterized ATPase subunits [14],[15]. In *Drosophila*, ISWI forms several kinds of chromatin remodeling complexes: ACF (ATP-utilizing chromatin assembly and remodeling factor) [16], CHRAC (chromatin accessibility complexes) [17], and NURF (nucleosome remodeling factor) [15]. In vitro assays have shown that these ISWI complexes facilitate the sliding of histone octamers on DNA [18]–[20]. The Remodeling and Spacing Factor (RSF) complex has been purified from human cells [21], and consists of two subunits: Rsf-1 and SNF2H, a human counterpart of *Drosophila* ISWI. RSF possesses not only the chromatin remodeling activity, but also chromatin assembly activity in vitro. Rsf-1 assembles nucleosomes randomly with its histone chaperone activity. The resulting nucleosomes are then redistributed into a periodic array by the ATP-utilizing nucleosome mobilization factor SNF2H [22]. No other single factor can execute the same activities as RSF. While the biochemical property of RSF has been investigated in vitro, the in vivo function of the factor remains elusive.

We investigated the biological function of RSF by using a genetically amenable organism *D. melanogaster*. Because the catalytic subunit ISWI is shared with other remodeling factors, we focused on the Rsf-1 subunit. The *Drosophila* genome contains one candidate gene *CG8677* (*Drosophila Rsf-1*: *dRsf-1*) [23],[24]. We show here physical interactions between dRsf-1 and ISWI suggesting that they function together as an RSF complex in vivo. A *dRsf-1* mutation dominantly suppresses position effect variegation (PEV). These results suggest that dRsf-1 functions in transcriptionally silent chromatin formation. Furthermore, we show physical and functional interactions of dRsf-1 with H2Av and the H2Av-exchanging factor Tip60 complex. Based on these findings, we propose that RSF plays a role in histone H2Av replacement, thereby contributing to the formation of silent chromatin structure.

## Results

### Characterization of dRsf-1

**Figure 1:**
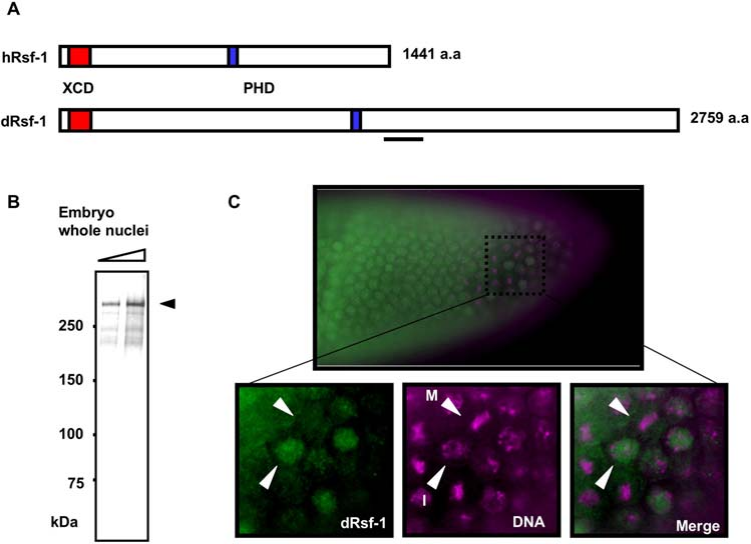
Characterization of dRsf-1. (A) Schematic comparison between human and *Drosophila* Rsf-1. These proteins contain the XCD (red box) and PHD (blue box) in the same order. The bar indicates the region against which antibodies were raised. (B) Immunoblot of embryonic nuclear proteins probed with affinity-purified anti-dRsf-1 antibodies. The antibodies specifically recognize a 300 kDa dRsf-1 protein. (C) Staining of embryos with the antibodies against dRsf-1 (green) and DAPI (magenta). Observed was uniform nuclear distribution of dRsf-1 (upper panel). Higher magnification of the embryonic cells (lower panel). White arrowheads show the interphase cell (I) and the metaphase cell (M).

The *Drosophila* genome contains a single candidate for human *Rsf-1/HBXAP* counterpart gene *CG8677* [23],[24]. The full-length cDNA of *CG8677* encodes a 2759 amino-acid protein, with the predicted molecular mass of 304 kDa. dRsf-1 protein contains a HBXAP conserved domain (XCD) at the N-terminus, and a plant homology domain (PHD) in the middle of the molecule (Figure 1A). While the homology between human Rsf-1 and *CG8677* gene product is restricted to XCD and PHD, these domains are found in the same order and *CG8677* is a sole gene encoding both these domains highly homologous to those in human Rsf-1 (Figure S1). Moreover, *CG8677* product is an acidic protein (pl 5.1) sharing several long stretches of acidic amino acids with human Rsf-1. Based on these data, we considered *CG8677* as *Drosophila Rsf-1* (*dRsf-1*). A search for Rsf-1 homologs in other species revealed candidate genes in mouse, zebra fish, and sea urchin [25], but not in yeast and plants (data not shown).

To gain insight into dRsf-1 cellular function, we raised antibodies against a region unique to the dRsf-1 protein (278 amino acid residues underlined in Figure 1A, bottom line). The affinity-purified antibodies specifically recognized approximately 300-kDa dRsf-1 proteins from whole nuclei of *Drosophila* embryos by western blotting (Figure 1B). Using the antibodies against dRsf-1, we first analyzed the localization of dRsf-1 in early *Drosophila* embryos and found that dRsf-1 is expressed ubiquitously (data not shown). During interphase, dRsf-1 localized to nuclei and diffused in the cytoplasm during metaphase (Figure 1C). A similar behavior has been reported for human Rsf-1 during cell division [23].

### dRsf-1 Localizes to Non-Transcribed Regions of Euchromatin

**Figure 2:**
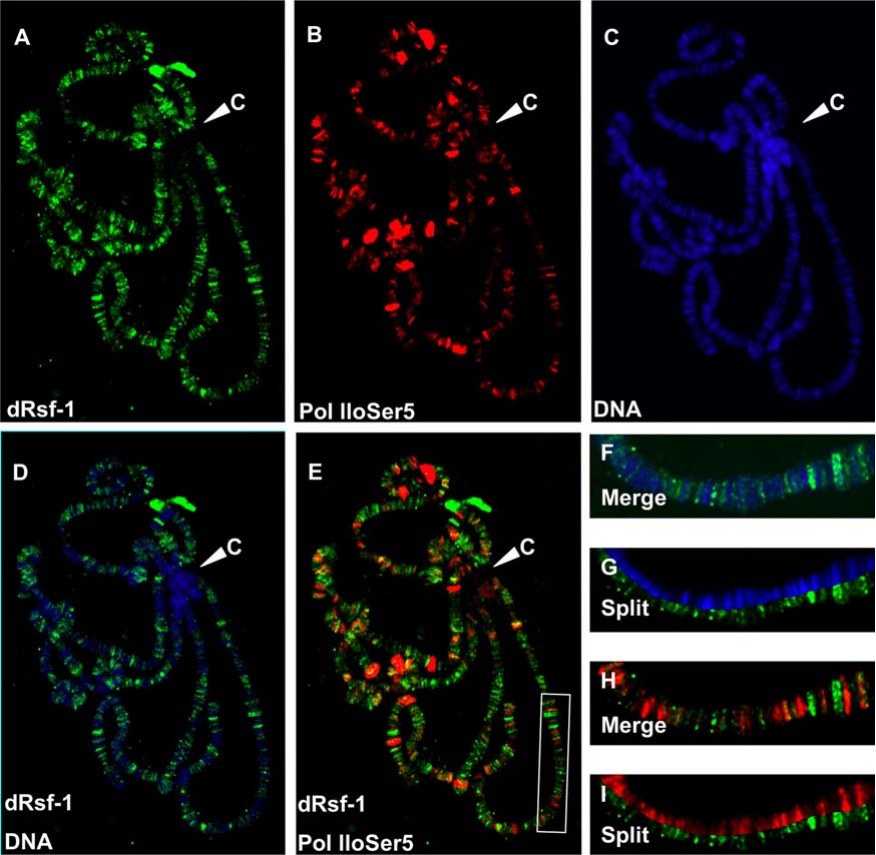
Localization of dRsf-1 protein on polytene chromosomes. (A–C) Distribution of dRsf-1 protein (green), Ser5-pohosohorylated Pol ll (red) and DNA stained with DAPI (blue) on wild-type polytene chromosomes. Merged and split images are shown in (D,F,G): dRsf-1 and DNA, and in (E,H,I): dRsf-1 and Pol llo^ser5^. dRsf-1 was primarily associated with interband regions, but not transcribed regions. No or very few dRsf-1 were associated with chromocenter (C). (F–I) Higher magnifications of the chromosome (white rectangle in (E)) are shown.

To directly visualize dRsf-1 localization on chromatin, we examined its distribution on salivary gland polytene chromosomes. We observed that dRsf-1 localizes to multiple sites along the polytene chromosomes (Figure 2A). The vast majority of these sites reside in interbands, regions of less condensed DNA stained lightly with DAPI (Figure 2D, 2F, and 2J). We were unable to detect dRsf-1 signals at the heterochromatic chromocenter (Figure 2D). To analyze a potential role of dRsf-1 in gene expression, we compared its distribution on polytene chromosomes with that of RNA polymerase ll (Pol ll). We used an antibody against the phosphorylated Ser5 of the Pol ll CTD (Figure 2B). The Ser5-phosphorylated Pol ll is present preferentially in promoter proximal regions and represents a form engaged in transcription [26]. dRsf-1 and Ser5-phosphorylated Pol ll showed predominantly non-overlapping distributions (Figure 2E, 2H, and 2I). These observations demonstrate that dRsf-1 localizes to non-transcribed regions of euchromatin.

### dRsf-1 Interacts with ISWI

**Figure 3:**
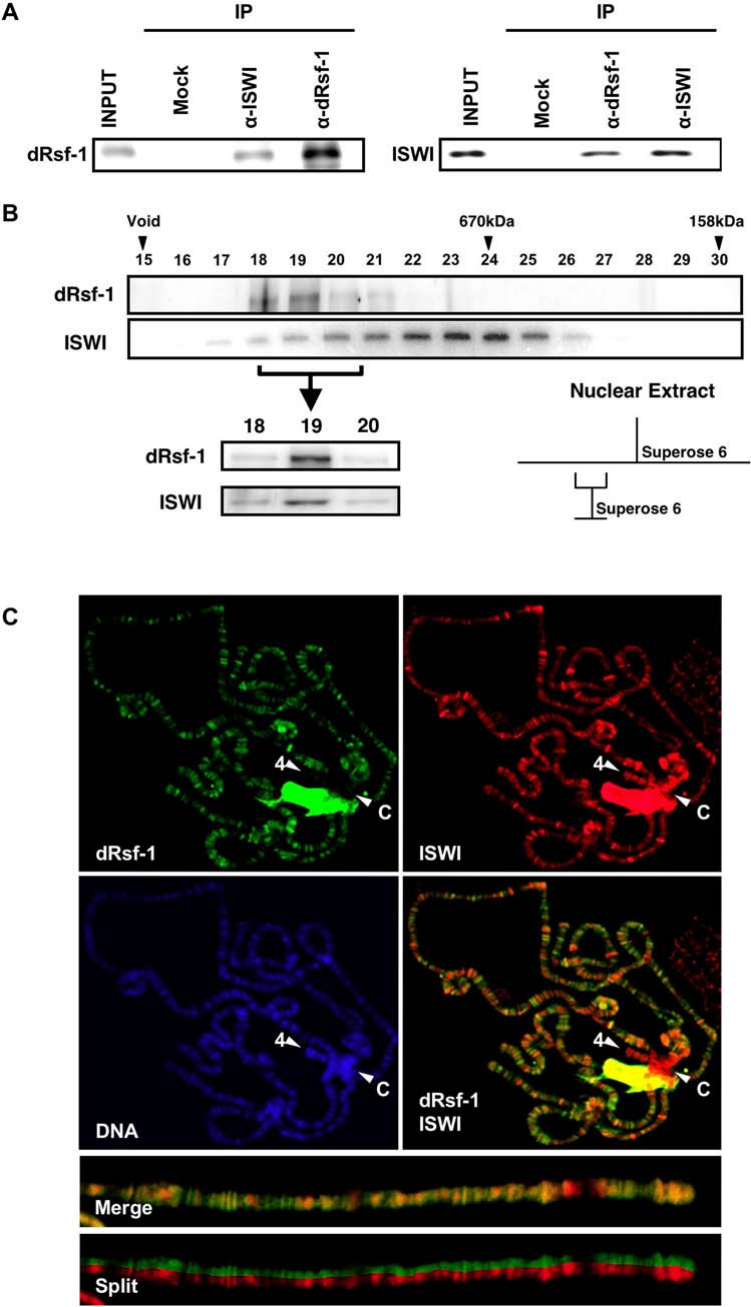
Interactions between dRsf-1 and ISWI. (A) Co-immunoprecipitation of dRsf-1 and ISWI. *Drosophila* embryonic nuclear extracts were subjected to immunoprecipitation using the anti-ISWI antibodies (left panel), anti-dRsf-1 antibodies (right panel) or control IgG (Mock). Bound proteins were separated by SDS-PAGE and analyzed by western blotting. Input represents 10% of the starting extract used for the immunoprecipitation. (B) Fractionation of dRsf-1 and ISWI by Superose-6 gel filtration. Upper panel: Gel filtration of an embryonic nuclear extract. Lower panel: the second gel filtration of dRsf-1 containing fractions (No.18–20 of the 1^st^ gel filtration). Both were assayed by antibodies against dRsf-1 and ISWI. Right panel: The purification scheme for *Drosophila* RSF. Arrowheads indicate void and elution volumes of molecular standards. While an expected size of a dRsf-1-ISWI hetero-dimer was approximately 450 KDa, dRsf-1 was eluted in a single peak around 1MDa, suggesting the presence of other interacting proteins. (C) Distribution of dRsf-1 protein (green), ISWI protein (red) and DNA stained with DAPI (blue) on wild-type polytene chromosomes. The merged image is shown in (dRsf-1 and ISWI). dRsf-1 and ISWI colocalize at almost all the euchromatic regions but not at the heterochromatic regions such as chromocenter (C) and the fourth chromosome (4). The large region near the chromocenter is nucleolus, which is known to show non-specific staining with various antibodies. Higher magnification of distal region of chromosome 2R was shown in lower pictures.

Human Rsf-1 forms the RSF complex with ISWI-type ATPase SNF2H [21]–[23]. Both subunits are essential for the activities of the complex in vitro [23]. To investigate whether the RSF complex is conserved in *Drosophila*, we first made co-immunoprecipitation analyses using embryonic nuclear extracts. ISWI proteins were co-immunoprecipitated with anti-dRsf-1 antibodies, but not with control IgG (Figure 3A, left panel). Reciprocally, we verified that dRsf-1 was co-immunoprecipitated with anti-ISWI antibodies (Figure 3A, right panel). Both types of the co-immunoprecipitation were observed in the presence of 80 µg/ml ethidium bromide, suggesting that the interaction between dRsf-1 and ISWI is not mediated through nucleic acids (Figure S2). Quantification of the band intensities indicated that almost all dRsf-1 interacts with ISWI in the embryonic nuclear extracts. Next, we fractionated an embryonic nuclear extract by gel filtration through Superose-6 and analyzed the fractions for the presence of dRsf-1 and ISWI by western blotting. dRsf-1 was eluted in a single peak at about 1MDa (Figure 3B). The majority of ISWI peaked around 670 KDa as previous report [18], which may correspond to ACF/CHRAC-containing fraction [17]. However, approximately 20% of ISWI was eluted wit the dRsf-1 peak. To test if dRsf-1 forms a complex with ISWI, we pooled the dRsf-1 peak fractions (No. 18–19), and again fractionated it by gel filtration through Superose-6. This process eliminated the ACF/CHRAC complex containing ISWI, and we observed that dRsf-1 and ISWI were eluted in a single peak. Finally we compared the distribution of dRsf-1 with that of ISWI on polytene chromosomes (Figure 3C). The distribution of dRsf-1 was mostly coincident with that of ISWI in euchromatic regions. Consistent with our co-immunoprecipitation data and the fact that ISWI is also included in other complexes, not all signals of ISWI colocalized with the dRsf-1 signals on polytene chromosomes. Indeed, we observed localization of ISWI but not dRsf-1 on the centromeric heterochromatin and the 4^th^ chromosome region (Arrow heads C and 4 in Figure 3B, respectively). These biochemical and cytological data indicate that ISWI and dRsf-1 work on chromatin as an RSF complex and also suggest that at least some ISWI-containing complexes are present on chromatin independently.

### Generation and Characterization of a *dRsf-1* Mutant

**Figure 4:**
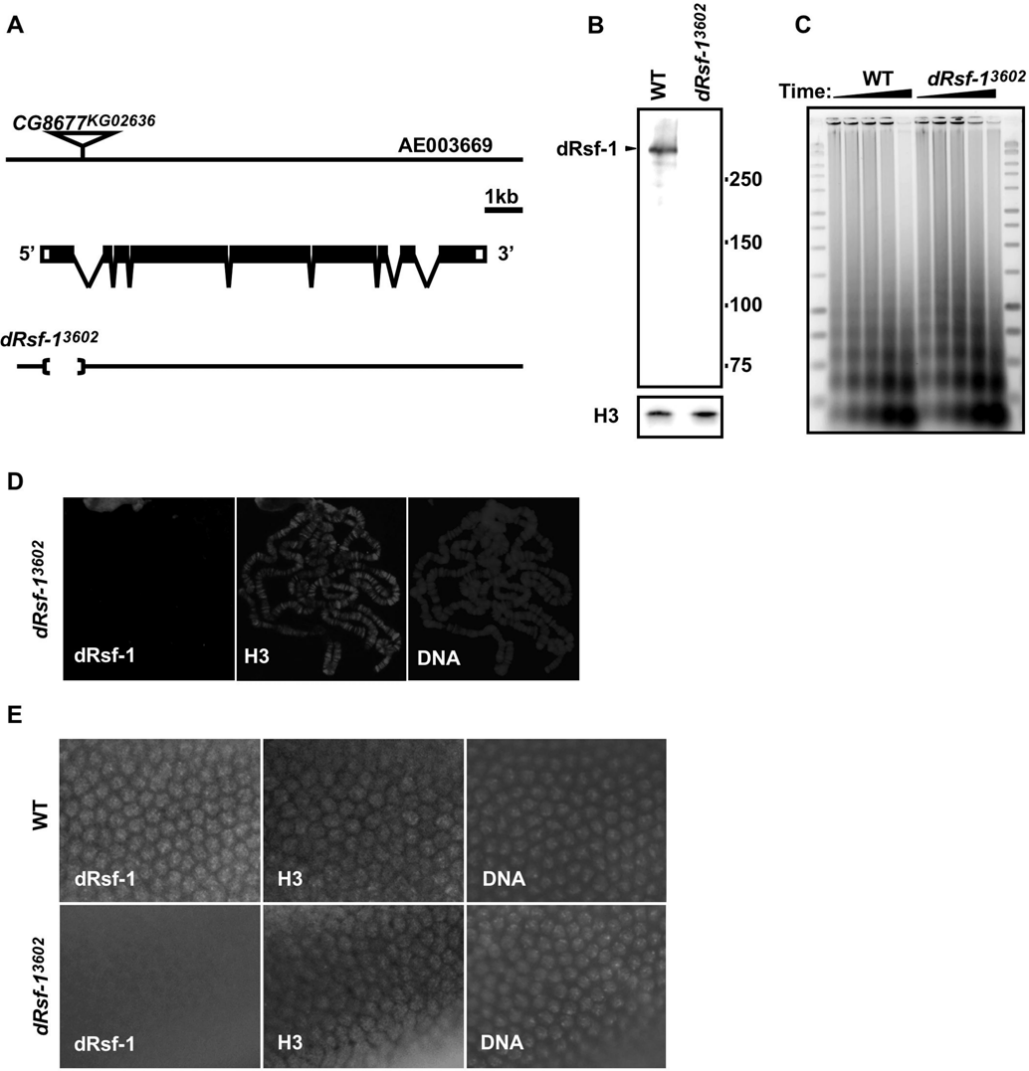
Generation of a dRsf-1 mutant. (A) Structure of the *dRsf-1* gene and position of the P-element insertion in *I(2)KG02636.* The allele *Df(2L)dRsf-1^3602^* (*dRsf-1^3602^*) obtained by an imprecise excision has an 876 bp deletion eliminating the translational initiation codon, the first exson and a part of the first intron of *dRsf-1* gene. (B) Lack of dRsf-1 protein in *dRsf-1^3602^*. Western blots of nuclear proteins from wild-type (WT: Oregon R) and homozygous *dRsf-1^3602^* embryos were probed with the affinity-purified anti-dRsf-1 antibodies; the anti-histone H3 antibodies were used to monitor the protein loading. dRsf-1 proteins were undetectable in the homozygous *dRsf-1^3602^* embryos. (C) MNase assay for analysis of chromatin structure in vivo. Nuclei from mixed stage embryos (10–22 h AEL) were incubated with increasing reaction times of MNase. The DNA was purified and separated on agarose gel and stained with SYBR green. (D) Staining of polytene chromosomes from a *dRsf-1* mutant larva with the anti-dRsf-1 antibodies and with DAPI. No staining with the anti-dRsf-1 antibodies was detected, while the over-all morphology or banding pattern appeared normal in these chromosomes. Chromosomes were stained with anti-histone H3 antibodies as a control. (E) Staining of an early embryo from wild type (WT) or the *dRsf-1* mutant. In the *dRsf-1* mutant embryo, we were unable to detect the signals in the nuclei with anti-dRsf-1 antibodies. Embryos were stained with anti-histone H3 antibodies as a positive control of immunostaining.

*dRsf-1* is mapped to the cytological interval 39B3-4 in the left arm of chromosome 2. The fly line *I(2)KG02636* carries a P-element *P{SUPor-P}* in the first intron of the *dRsf-1* gene (Figure 4A). Homozygous *I(2)KG02636* animals are viable and the insertion does not affect the *dRsf-1* transcription (data not shown). To assess the in vivo function of dRsf-1, we generated *dRsf-1* deletions by imprecise excision of the P-element in *I(2) KG02636*. Sequence analysis of the resulting allele, *Df(2L)dRsf-1^3602^* (*dRsf-1^3602^*), confirmed an 876-bp deletion that eliminates the translational initiation codon, the first exon and a part of the first intron of *dRsf-1* gene (Figure 4A). dRsf-1 protein was not detectable in homozygous *dRsf-1^3602^* embryos by western blotting (Figure 4B). No staining with anti-dRsf-1 antibodies was observed on polytene chromosomes of mutant larvae (Figure 4D) and the nuclei of early embryos from the mutant flies (Figure 4E). From these data, *dRsf-1^3602^* appears to be a null allele of *dRsf-1*.

Although evolutionary conservation of Rsf-1 implies a critical role in development, almost all *dRsf-1^3602^* homozygous flies are viable. Only about 10% of the homozygous *dRsf-1^3602^* flies died in the pupal stage. We observed a melanotic tumor phenotype in these animals (data not shown). The loss of *dRsf-1* does not affect the gross appearance of polytene chromosomes (Figure 4D) and we were unable to observe any change in the nucleosome repeat length of bulk chromatin derived from *dRsf-1^3602^* embryos compared with wild type embryos (Figure 4C). Consistent with our observations, recent study has also reported that expression of dominant-negative ISWI protein did not affect the nucleosome spacing and sensitivity to micrococcal nuclease digestion [27]. As the immunofluorecence analysis shows that dRsf-1 localizes on the limited regions on chromatin (Figure 2A), it may be difficult to observe the effect of *dRsf-1* mutation on the bulk chromatin structure. Homozygous *dRsf-1^3602^* adult flies are fertile and phenotypically normal. These results indicate that dRsf-1 is not essential for viability and fertility. We used homozygous *dRsf-1^3602^* flies in subsequent experiments.

### Suppression of Position Effect Variegation in *dRsf-1* Mutant Flies

**Figure 5:**
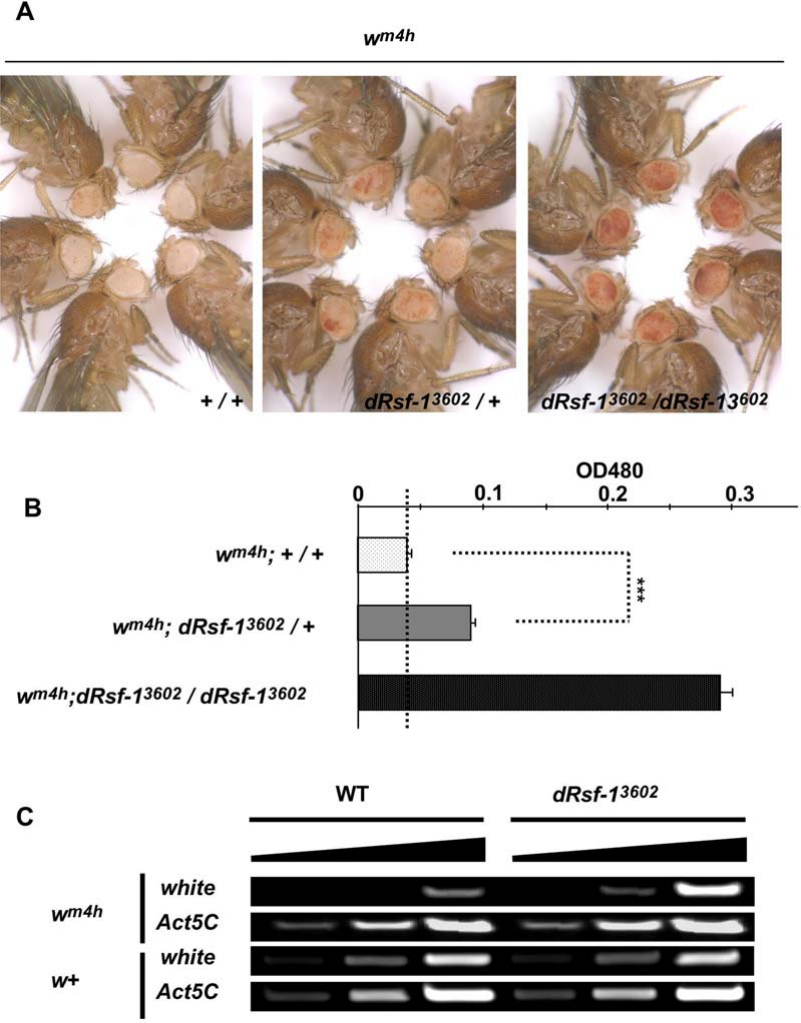
Suppression of position effect variegation by the *dRsf-1* mutant. (A) Eye phenotypes of flies with *In(1)w^m4h^* in *dRsf-1* wild type (left panel), heterozygous *dRsf-1^3602^* (middle panel) and homozygous *dRsf-1^3602^* (right panel) genetic backgrounds. The dRsf-1 mutant flies show dominant suppression of PEV and homozygous *dRsf-1^3602^* flies exhibit strong suppression of PEV. Males were photographed one day after eclosion. (B) Measuring of eye pigmentation in the indicated lines. Error bars represent SD throughout the paper. Asterisks indicate statistical significance: (***) *P*<0.001. (C) Expression levels of *white* and *Act5C* were examined by RT-PCR. The amounts of *white* transcripts were actually increased in the *dRsf-1^3602^* homozygous flies in *w^m4h^*, but not in *w^+^*.

Judging from the in vitro activities of human RSF, we expected that dRsf-1 should play a role in the regulation of chromatin structure in vivo. To test this possibility, we examined the effect of the *dRsf-1* mutant allele, *dRsf-1^3602^*, on PEV. A series of mutations that suppress or enhance PEV have been identified so far, and most of the corresponding genes have been shown to be involved in the regulation of chromatin structure [28],[29]. The *In(1)w^m4h^* allele is caused by an inversion that positions the *white* gene next to the centromeric heterochromatin of the X chromosome [30],[31]. This leads to a phenotype in which the eye color is a mosaic of red and white. As shown in Figure 5A, the variegation was significantly suppressed in animals heterozygous for the *dRsf-1^3602^* allele, with red-pigmented regions significantly increased compared to *In(1)w^m4h^* alone (Figure 5A and 5B). Moreover, the variegation was suppressed more strongly in homozygous *dRsf-1^3602^* animals; with most regions of their eyes exhibiting red pigmentation (Figure 5A, right panel). Essentially the same results were also observed in flies doubly heterozygous for *dRsf-1^3602^* and the deficiency *Df(2L)TW1* or *Df(2L)DS6*, which both lack the *dRsf-1* gene region (Figure S3). Quantitative analyses revealed that the amounts of the red pigment were increased ∼2-fold and 6-fold in the *dRsf-1^3602^* heterozygous and homozygous flies, respectively (Figure 5B). Moreover, reverse transcription (RT)-PCR analysis revealed that the amounts of *white* transcripts from *w^m4h^* allele, but not from *w^+^* were actually increased in the *dRsf-1^3602^* homozygous flies (Figure 5C). This shows that *dRsf-1* does not directly regulate the transcription of *white* gene on the normal chromosomal position. These data demonstrate that *dRsf-1^3602^* allele is a dominant suppressor of PEV and indicate that dRsf-1 is involved in heterochromatin-induced silent chromatin formation.

### dRsf-1 Contributes to H2Av Incorporation in the Pathway of Silent Chromatin Formation

**Figure 6:**
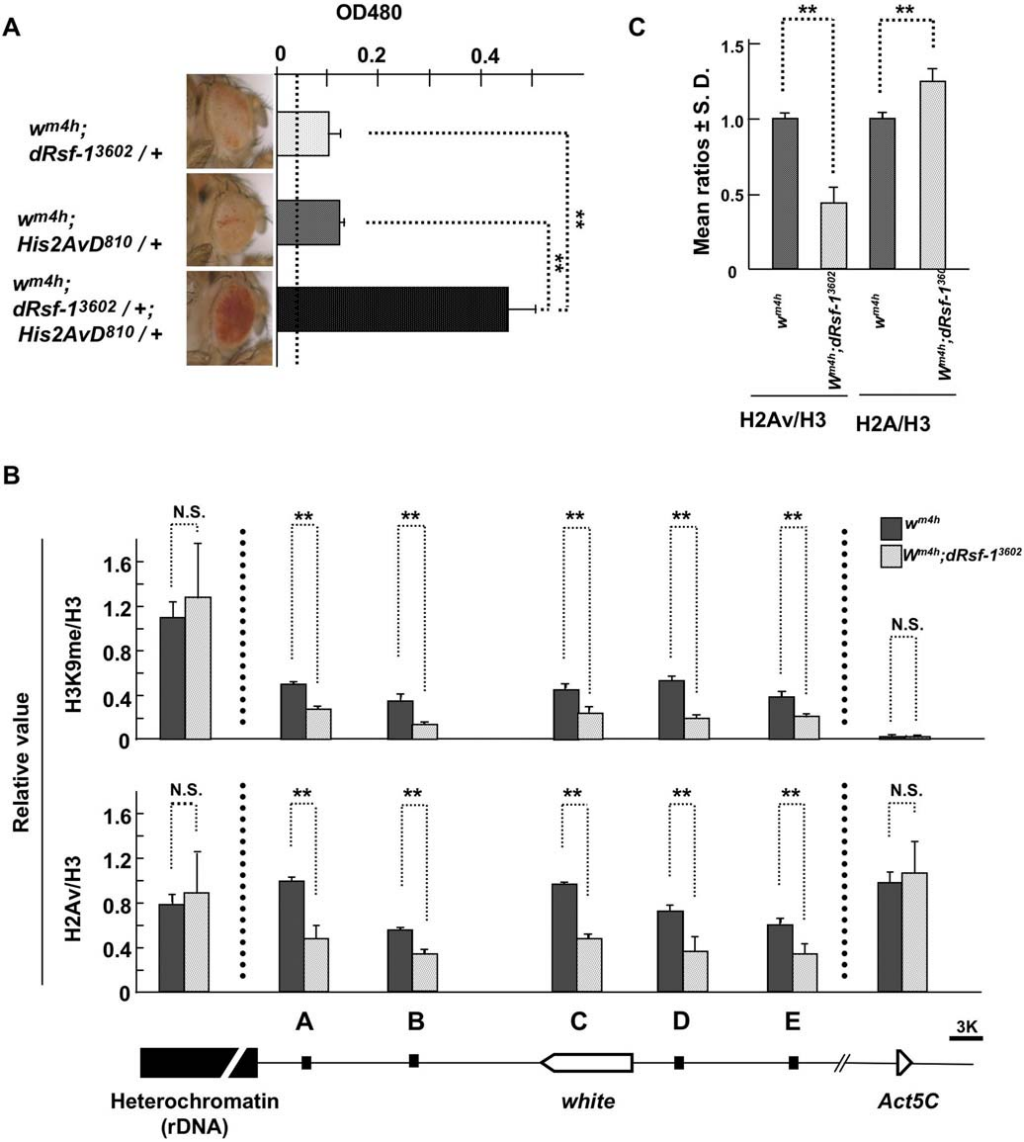
Effect of dRsf-1 mutation on silent chromatin formation. (A) Genetic interaction between dRsf-1 and H2Av. dRsf-1 or H2Av mutation dominantly suppressed PEV weakly and double heterozygote of these mutations strongly suppressed PEV resulting in red eyes as shown in photos of male eye (under the graph). The pigments extracted from male heads were measured at 485 nm. Error bars indicate standard deviations from independent four measurements (shown in the graph). Vertical dotted line shows the value for the control fly (*w^m4h^; +/+*). (B) ChIP analyses with the anti-dmH3K9 antibodies (upper panel) and anti-H2Av antibodies (lower panel) in *In(1)w^m4h^* and *In(1)w^m4h^;dRsf-1^3602^* embryos. The level of dmH3K9 or H2Av enrichment was normalized to global nucleosomal occupancy measured by histone H3 enrichment. The value of *y*-axis represents the ratio of immunoprecipitated DNA with anti-dmH3K9 antibodies or anti-H2Av antibodies to that with anti-H3 antibodies. A diagram of the rearranged *white* locus on X chromosome is shown in the bottom. Asterisks indicate statistical significance: (**) *P*<0.05. (N.S.) Not significant.

Recent studies have shown that the histone H2A variant H2Av forms a sequential pathway for repressive chromatin assembly [13]. To test whether dRsf-1 is involved in this pathway, we tested for a functional interaction between RSF and H2Av. While both *dRsf-1^3602^* and *H2Av^810^* heterozygous mutants suppressed PEV weakly (Figure 6A) [13], quantification of the red eye pigment showed a synergistic increase in the double heterozygotes compared to each single heterozygote (Figure 6A). The observed genetic interaction between *dRsf-1* and *H2Av* suggests a role for RSF in the H2Av-mediated pathway of silent chromatin formation.

How is RSF involved in the assembly of silent chromatin? To gain clues about this question, we examined the chromatin status in the *dRsf-1^3602^* mutant by chromatin-immunoprecipitation (ChIP). Our major focus was on the rearranged *white* regions in *w^m4h^*. In addition, we analyzed an rDNA coding sequence in the β-heterochromatin [32],[33] and an actively transcribed *Act5C* region as controls. First, we analyzed histone H3K9 methylation that is a typical mark of the heterochromatin region. H3K9 methylation has been detected in the rearranged *white* region of *w^m4^* flies [34],[35]. We observed that the level of H3K9 di-methylation (dmH3K9) was clearly reduced by 30∼60% in the *dRsf-1^3602^* mutant flies as compared with *dRsf-1* wild type, while the level in the rDNA region was not changed significantly between the mutant and wild type (Figure 6B, upper graph). The level of dmH3K9 was extremely low and also was not changed in the *Act5C* coding region. Next, we investigated the upstream event of H3K9 methylation. The recruitment of the H2Av has been shown to be a necessary step prior to H3K9 methylation [13]. This led us to do the ChIP analysis using anti-H2Av antibodies. Notably we observed a significant decrease in the levels of H2Av around the rearranged *white* region in the *dRsf-1^3602^* mutant flies, while the levels of H2Av did not decrease in the rDNA and the actively transcribed *Act5C* regions (Figure 6B, lower graph). There is no significant difference in the mRNA levels of *H2Av* in the *dRsf-1^3602^* mutant flies compared with the control flies (Figure S4), indicating that the observed decrease is not due to reduced expression of *H2Av* in *dRsf-1^3602^* mutant flies. Furthermore, the levels of H2A increased in the region (region “A” in Figure 6B) that showed an H2Av decrease (Figure 6C), indicating that the observed decrease in H2Av is due to less frequent replacement of H2A by H2Av. These results of ChIP analyses indicate that RSF contributes to H2Av incorporation and also suggest that RSF is not directly involved in the formation of heterochromatin but necessary for heterochromatin spreading into neighboring regions.

### Interactions between dRsf-1 and H2Av

**Figure 7:**
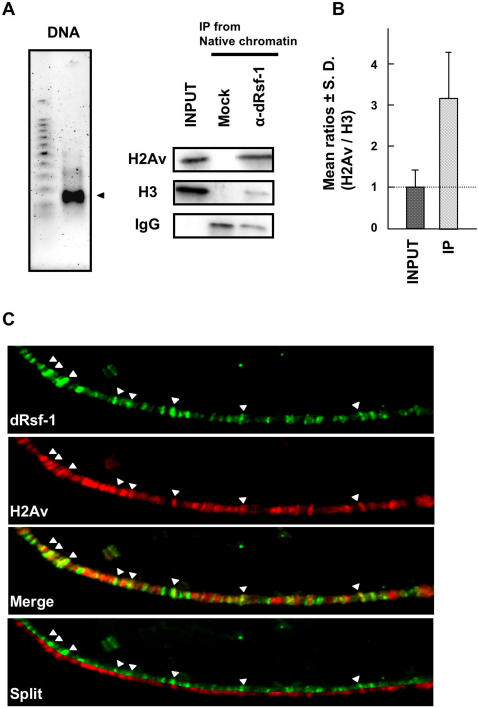
The biochemical and cytological interactions between RSF and H2Av. (A) Co-immunoprecipitation of RSF and H2Av. Native chromatin samples were isolated from embryonic nuclei by MNase digestion. The DNA was purified and separated on agarose gel and stained with SYBR green (Left panel: Size was determined by reference to a 100-bp ladder. Arrowhead indicates mono-nucleosome). Native chromatin samples were subjected to immunoprecipitation using anti-dRsf-1 antibodies or control IgG (Mock). The proteins were separated by SDS-PAGE and analyzed by western blotting with anti-H2Av antibodies and anti-histone H3 antibodies as a control. Input represents 2% of the starting extract used for the immunoprecipitation. (B) Semi-quantitative analyses of interaction between dRsf-1 and H2Av proteins. The amount of H2Av proteins in nucleosomes immunoprecipitated with anti-dRsf-1 antibodies (IP) was normalized by that of H3 proteins, and compared with the input proteins. The mean ratio of input proteins was set as 1. Error bars indicate standard deviations from independent three measurements. (C) dRsf-1 colocalizes with H2Av at multiple sites along polytene chromosomes. Immunostaining of chromosome 3R with anti-dRsf-1 antibodies (green) and anti-H2Av antibodies (red). Merged images and split images were shown on the bottom. Arrowheads point to strong dRsf-1 signals that colocalize with H2Av signals.

To address how RSF governs H2Av incorporation, we analyzed interaction between RSF and H2Av using soluble nuclear extracts from embryos. However, we were unable to detect co-immunoprecipitation of H2Av with RSF. It is possible that RSF interacts with H2Av on chromatin but not in the soluble nuclear extract. Therefore, we examined the association of dRsf-1 with chromatin containing H2Av. Nuclei from the *Drosophila* embryos were digested with micrococcal nuclease to yield predominantly mono-nucleosomes (Figure 7A, left panel). When the nucleosomes were immunoprecipitated with anti-dRsf-1 antibodies, H2Av proteins were co-immunoprecipitated (Figure 7A, right panel). Furthermore, the H2Av proteins were enriched in the immunoprecipitated nucleosomes compared with the bulk nucleosomes (Figure 7B). To confirm the interaction between dRsf-1 and H2Av on chromatin, we examined whether dRsf-1 colocalizes with H2Av on larval polytene chromosomes. Although the distribution of dRsf-1 was not completely coincident with that of H2Av, strong immunofluorecence signals of dRsf-1 colocalized with H2Av in multiple sites on the polytene chromosomes (Figure 7C, arrow heads). The biochemical and cytological interactions between dRsf-1 and H2Av suggest that RSF functions at (or immediately upstream of) H2Av incorporation rather than playing an indirect role through other step(s).

### RSF Interacts with the Tip60 Complex

**Figure 8:**
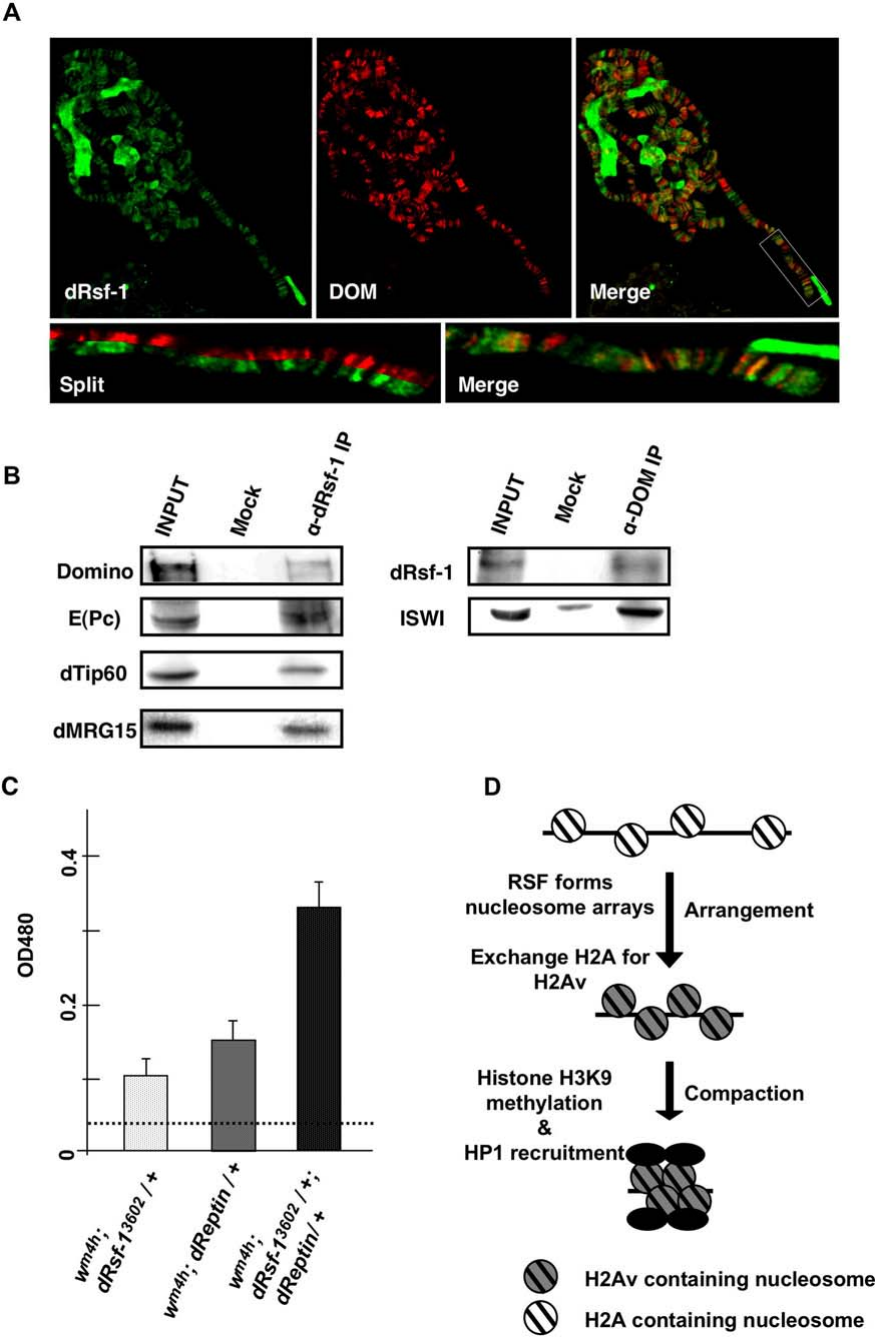
RSF interacts with Tip60 complex. (A) Distribution of dRsf-1 protein (green) and Domino (red) on wild-type polytene chromosomes. The merged image is shown in the right panel (Merge). Extensive overlap between dRsf-1 and Domino on chromosome 3R is evident in the enlarged merged and split images of the region encompassed by a white rectangle (lower panel). (B) Co-immunoprecipitation of RSF and Domino. *Drosophila* embryonic nuclear extracts were used for immunoprecipitation using anti-dRsf-1 antibodies (left panel), anti-Domino antibodies (right panel) and control IgG (Mock). The proteins were separated by SDS-PAGE and analyzed by western blotting. Several subunits of the Tip60 complex were co-immunoprecipitated with anti-dRsf-1 antibodies. RSF was co-immunoprecipitated with anti-Domino antibodies, but not with control IgG. Input represents 10% of the starting extract used for the immunoprecipitation. (C) Genetic interaction between *dRsf-1* and *dReptin*. The pigments extracted from male heads of each fly lines were measured at 485 nm. Error bars indicate standard deviations from independent four or three measurements (shown in the graph). Dotted line shows the value for the control fly (*w^m4h^; +/+*). (D) Proposed pathway for silent chromatin formation. RSF forms nucleosome arrays and contributes to the exchange of H2A with H2Av in an early step of the silent chromatin formation. These nucleosome arrays are stabilized by the internucleosome interactions through H2Av proteins. This triggers the following steps of the silent chromatin formation such as the recruitment of HP1 and the methylation of H3K9.

In yeast, the ATP-dependent chromatin remodeling complex SWR1 has been reported to exchange histone H2A with H2A.Z [36]. A *Drosophila* counterpart of the yeast SWR1 complex is the Tip60 complex that consists of multisubunits including Tip60, Domino, dMRG15 and dReptin, and can exchange phospho-H2Av with an unmodified H2Av [37]. Recent report has shown that the Tip60 complex can promote the generation of silent chromatin [38]. It is possible that RSF is functionally related to the Tip60 complex. To test the possibility, we first analyzed the distribution of dRsf-1 and Domino, a *Drosophila* counterpart of yeast *Swr1* [37],[39], on polytene chromosomes. As shown in Figure 8A, these proteins colocalize on many loci along the polytene chromosomes. While the signals were not completely coincident, it is most likely due to the multifunction of Domino; some of Domino is also involved in transcriptional activation [40]. We then analyzed physical interaction between RSF and Domino by immunoprecipitation assays using embryonic nuclear extracts. Domino proteins were co-immunoprecipitated with anti-dRsf-1 antibodies (Figure 8B). In a reciprocal test, dRsf-1 and ISWI proteins were co-immunoprecipitated with anti-Domino antibodies (Figure 8B). We also observed co-immunoprecipitation of other Tip60 complex components, E(Pc), dTip60 and dMRG15, with dRsf-1 (Figure 8B). Finally we analyzed a functional interaction between dRsf-1 and dReptin, a core subunit of the Tip60 complex. While both *dRsf-1^3602^* and *dReptin* heterozygous mutants suppressed PEV (Figure 8C) [38], their double heterozygotes showed a synergistic effect on the suppression of PEV (Figure 8C). This effect is not due to decreased expression of dReptin in the Rsf-1 mutant (Figure S4). Taken together these data suggest that RSF contributes to exchange of histone H2Av through association with the Tip60 complex.

## Discussion

In this study, we reported the biological function of the chromatin remodeling complex RSF in *D. melanogaster*. We generated a mutant allele of *dRsf-1, dRsf-1^3602^*, and found that it dominantly suppresses PEV. These data provide the first evidence that RSF plays a role in transcriptionally silent chromatin formation in vivo. Furthermore, our findings suggest that dRsf-1 functions at H2Av replacement in the pathway of silent chromatin formation.

### ISWI Complex RSF

Several kinds of ISWI-containing complexes have been shown in *Drosophila*. NURF, ACF and CHRAC have been identified using in vitro assays for nucleosome remodeling activities in embryonic extracts of *Drosophila* [15]–[17]. Toutatis (*Drosophila* ortholog of human TIP-5) ISWI complex has been reported to positively regulate gene activities [41]. We have herein identified another ISWI chromatin remodeling complex, RSF, in *Drosophila*.

All of *dRsf-1*, *nurf301* and *Acf1* mutations suppress PEV (this study) [42],[43]. The *nurf301* mutation might suppress PEV due to the up-regulation of the genes on the X chromosome [44]. The changes in bulk chromatin repeat length in the *Acf1* mutant flies could be ascribed to a defect in the incorporation of histone H1 [45], which suggests a function for ACF in heterochromatic regions. However, in *dRsf-1^3602^* mutant flies, we were unable to detect an abnormally decondensed X-chromosome as is observed in the *nurf301* mutant flies [44], nor a nucleosome spacing defect as has been detected in *Acf1* mutant flies [42]. Instead, the level of histone H2Av on chromatin is reduced in the *dRsf-1^3602^* mutant and our biochemical and genetic studies show that dRsf-1 contributes to silent chromatin formation coincident with histone H2Av incorporation in euchromatin regions. Judging from these observations, the effect of each mutation on PEV appears to come from different mechanisms. Various types of ISWI complex seem to exist independently on chromatin and execute different functions in vivo.

### Mutations of Non-Heterochromatic Proteins Suppress PEV

PEV is thought to be caused by spreading of the heterochromatic state into neighboring regions of euchromatin [46]. While products of some *Su(var)* genes are thought to localize and function on heterochromatin regions, proteins encoded by other *Su(var)* genes such as *dRsf-1* (this study), *E(Pc)* [47], *Z4* [48] and so on do not localize to heterochromatin regions. A recent study has reported that the mutation of *dG9a*, the *Drosophila* ortholog of euchromatic H3K9 methyltransferase *G9a*, also suppresses PEV [49]. Because, in *Drosophila*, localization of such proteins is usually analyzed on polytene chromosomes in the post mitotic stage, one possibility is that they bind to heterochromatin regions transiently in cell cycle progression or during an early developmental stage. For example, Su(var)3-3, a *Drosophila* homolog of LSD1 has been reported to localize on heterochromatin region during early embryonic stages and to play an essential role in establishment of heterochromatin [35]. However, we were unable to detect the dRsf-1 protein on heterochromatin regions during cell cycle progression or during early embryonic stages (data not shown).

Thus, a more plausible interpretation of PEV suppression in the *dRsf-1* mutant is that dRsf-1 activities on a juxtaposed euchromatic region can allow the spreading of centromeric heterochromatin in the *dRsf-1^+^* background. We speculate that PEV could occur through not only the invasion of heterochromatin, but also the guidance of centromeric heterochromatin spreading from a potential facultative heterochromatin region. Indeed, the formation of constitutive heterochromatin and facultative heterochromatin share common molecular mechanisms [50]. When the potential facultative heterochromatin region becomes juxtaposed with the constitutive heterochromatin region, the heterochromatic state might be easily spread into the neighboring region.

### RSF Functions in an Early Step of the Silent Chromatin Formation

We found that the level of H2Av on chromatin was reduced in *dRsf-1* mutant flies (Figure 6). We also observed the physical and functional interactions of RSF with H2Av and the H2Av-exchanging factor Tip60 complex (Figures 7 and 8). These findings raise an intriguing possibility that RSF directs local H2Av replacement through its association with the Tip60 complex during the silent chromatin formation. Our future study should address whether the genetic interaction between *dRsf-1* and Tip60 complex component *dReptin* results in a defect in H2Av deposition at the *white* locus close to heterochromatin.

Although the level of H2Av on chromatin was reduced in *dRsf-1* mutant, we could still detect a residual level of H2Av, suggesting that some factor other than RSF may also contribute to the step. The presence of such factor may also explain our observation that the gross distribution of Domino along polytene chromosomes was not altered in the dRsf-1 mutant (unpublished observation). Loss of both RSF and the putative factor may be required to cause a dramatic change in the localization of Domino. The presence of other factor may also explain why *H2Av* is essential for fly development [51] but *dRsf-1* is not. Furthermore, while dRsf-1 is absent from the chromocenter (Figure 2D), H2Av has been reported to be a component of centromeric chromatin [12]. Therefore, another remodeling complex or mechanisms must be responsible for the deposition of H2Av at constitutive heterochromatin.

Interestingly, H2A.Z, the H2Av ortholog in other organisms, has been reported to affect chromatin condensation in vitro [52]. The acidic patch of H2A.Z has been thought to allow strong interaction with N-terminal tail of H4 from a neighboring nucleosome [53]. The nucleosome arrays formed by RSF may create a platform for incorporation of H2Av and also allow the precisely arranged nucleosome-nucleosome interactions through H2Av. As incorporation of yeast H2A.Z, Htz1, into nucleosomes inhibits chromatin remodeling activities [54], it may be necessary to remodel the basal nucleosome array before incorporation of h2Av. The resulting nucleosome array may easily promote HP1-mediated higher-order chromatin folding as observed for H2A.Z-containing nucleosome array [52], and facilitate H3K9 methylation.

Based on our findings and the above information, we propose the following pathway of condensed silent chromatin formation (Figure 8D): First RSF forms nucleosome arrays and induces exchange of H2A for H2Av through association with the Tip60 complex. This allows further steps of silent chromatin formation, such as histone H3K9 methylation and HP1 binding, to proceed or proceed more efficiently, which ultimately lead to chromatin compaction.

## Materials and Methods

### Fly Strains and Genetics

Flies were raised on a standard agar/cornmeal/yeast medium. Stocks and crosses were maintained at 25°C. The *CG8677^KG02636^* and *In(1)w^m4h^* flies were obtained from Bloomington Stock Center. The *His2Av^810^/TM3* were from Drosophila Genetic Resource Center (DGRC). The *dReptin/TM3* was a gift from Mattias Mannervik. The *CG8677^KG02636^* insertion line contains a P{SUPor-P} transposon within the first intron of *dRsf-1*. The transposon was mobilized in the presence of the P transposase Δ2–3. Total of about 1000 lines were analyzed by PCR to detect deletion across the *dRsf-1* locus.

### Position Effect Variegation Assays

To measure eye pigmentation, 10 male heads (24 hours post eclosion) from each line were homogenized in 200 µl 1∶1 mixture of chloroform/0.1% ammonium hydroxide. The homogenates were centrifuged and removed 100 µl of the upper aqueous layer to measure absorbency at 480nm [55]. Four or three independent samples were measured for each line.

### Antibody Generation

The DNA fragment encoding amino acid residues 1443–1719 of dRsf-1 polypeptide was cloned into a pET28a vector (Novagene). The protein fragments expressed in *E. coli* BL21 (DE3) RIL at 37°C were purified by an affinity chromatography on the Ni-NTA resin (Sigma) according to the manufacture's protocol and used to generate polyclonal antibodies in rabbits. Crude antiserum was purified by an affinity chromatography using the antigen polypeptides coupled to CNBr-activated Sepharose 4B (Amersham Biosciences). All immunological procedures were performed with the affinity purified anti-dRsf-1 antibodies.

### Western Blotting

Western blot analyses were performed using a standard protocol. The nuclei were prepared from embryos (collected 10–22 hours after egg laying: AEL) as described previously [56]. Nuclear proteins were separated by SDS-PAGE, transferred to a PVDF membrane (Roche), probed with the rabbit antibodies against dRsf-1, ISWI, H2Av, and Domino [12],[39],[57], followed by horseradish peroxidase-linked anti-rabbit IgG and detected using SuperSignal (Pierce).

### Immunostaining of Polytene Chromosomes

Salivary gland chromosomes from third instar larvae were fixed for 2 minutes in 45% acetic acid/1.85% formaldehyde and stained as descried previously [58]. The anti-dRsf-1 antibodies were used at a 1/100 dilution. Other antibodies used in this study include rabbit antibodies against ISWI, mouse antibody against Pol IIo^ser5^ (Covance) and rabbit antibodies against H2Av [12] and rat antibodies against Domino [37]. Secondary antibodies used were Alexa fluor™ 488 anti-rabbit IgG (Molecular Probes), Cy3 anti-mouse IgM and Cy3 anti-rat IgG (Jackson ImmunoResearch). For double staining, HiLyte Fluor™ 488 Dye and HiLyte Fluor™ 555 Dye (ANASPEC) were conjugated to anti-dRsf-1 and anti-ISWI or anti-H2Av antibodies, respectively. The labeling was done according to the manufacturer's protocol. Finally, the samples were mounted using a VECTASHIELD mounting medium containing DAPI (Vector Laboratories).

### Protein Biochemistry

The nuclei were extracted for 30 minutes in a nuclear extraction buffer (20 mM Tris-HCl, pH7.9, 1.5 mM MgCl2. 0.2 mM EDTA, 0.2 mM EGTA, 400 mM NaCl, 25% glycerol) in the presence of a protease inhibitor cocktail (Sigma). Antibodies were coupled to Protein-G Agarose (Upatate) for 6 hours and incubated with the nuclear extracts containing approximately 200 µg of proteins in an immunoprecipitation buffer (20 mM Tris-HCl, pH7.9, 0.5 mM EDTA, 150 mM NaCl, 10% glycerol) for 3 hours at 4°C. Subsequently beads were washed five times with a wash buffer (20 mM Tris-HCl, pH7.9, 0.5 mM EDTA, 150 mM NaCl, 0.01% NP-40, 10% glycerol).

Native chromatin samples were prepared as described [59] from *Drosophila* embryonic nuclei. Antibodies were coupled to Protein-G Agarose (Upstate) and the beads were washed five times with wash buffer (20 mM Tris-HCl, pH7.9, 0.5 mM EDTA, 300 mM NaCl, 0.05% NP-40, 10% glycerol). Bound proteins were eluted with an SDS-sample buffer (0.1 M Tris-HCl, pH6.8, 5% SDS, 0.1 M DTT, 20% glycerol), and resolved by SDS-PAGE and detected on Western blots.

For gel filtration, nuclear extract proteins (2 mg) were fractionated on a Superose 6 HR 10/30 FPLC column (GE) equilibrated with high salt-extraction buffer (20 mM Tris-HCl, pH7.9, 0.5 mM EDTA, 0.5 mM EGTA, 500 mM NaCl, 10% glycerol). Fractions (0.5 ml) were collected and analyzed by western blotting. Before the second gel filtration, dRsf-1-containing fractions (No. 18–20) were pooled and concentrated by Amicon Ultra (MILLIPORE).

### Chromatin Immunoprecipitation

ChIP assays were performed as described [30]. Anti-histone H3 (GeneTex), anti-histone H2Av [12], anti-histone H2A [12] and anti-histone H3K9 dimethyl (Upstate) antibodies were used for ChIP assays. The precipitated DNA fragments were amplified by PCR using Blend Taq (TOYOBO). The primer pairs were used to amplify ∼100-base pair fragments are described in Table S1. The PCR products were resolved by gel electrophoresis, stained with SYBR green (Molecular Probes), and analyzed by a LAS-1000 luminescent image analyzer (FUJIFILM). Averages and SDs (standard deviations) from at least three independent experiments are shown in the figures.

### RT-PCR

Total RNA was isolated using Sepazol RNA1 (Nakarai) from 50 mg of mixed-stage embryos (10–22 h AEL). The RNA was then treated with RNase-free DNase l (TaKaRa). cDNA was synthesized using a 1^st^ Strand cDNA Synthesis Kit for RT-PCR (Roche) according to the manufacturer's protocol. The cDNA was amplified by PCR and analyzed as described [34].

### In Vivo Chromatin Structure Analysis

Chromatin structure was examined by analyzing the pattern of the products partially digested with MNase. MNase assays were carried out as described [60]. DNA samples were size separated on 2% TAE agarose gel, and stained with SYBR green (Molecular Probes), and analyzed by the LAS-1000 luminescent image analyzer (FUJIFILM).

## Supporting Information

Figure S1 Conservation of PHD fingers between Drosophila CG8677 and human Rsf-1.
Amino acid identities in PHD fingers were compared between various Drosophila and human proteins. Upper panel: Among Drosophila proteins, the PHD finger in CG8677 exhibits the highest homology with that in hRsf-1. Lower panel: Among human proteins, the domain in hRsf-1 shows the highest homology with that in CG8677.
(2.45 MB TIF)

Figure S2 Co-immunoprecipitation of dRsf-1 with ISWI from embryonic nuclear extract in the presence of ethidium bromide (EtBr).
Immunoprecipitation was carried out with anti-dRsf-1 antibodies, anti-ISWI antibodies or control IgG; subsequently western blotting of the immunoprecipitates was probed with anti-ISWI antibodies or anti-dRsf-1 antibodies. To prevent of nucleic acids-dependent protein interactions, the extract was incubated with 80 µg/ml EtBr on ice for 1 hr before the immunoprecipitation. Input represents 10% of the starting extract used for the immunoprecipitation.
(0.79 MB TIF)

Figure S3 Comparison of the phenotype between dRsf-13602 and deficiencies lacking the dRsf-1 locus.
(A) Schematic representation of the cytological region 38-39. Df(2L)DS6 deletes the 38E2-39E7 region and Df(2L)TW1 deletes the 38A7-39C3 region.
(B) The double heterozygotes of dRsf-13602 and deficiency Df(2L)DS6 or Df(2L)TW1 suppressed the variegation strongly in a similar manner as the dRsf-13602 homozygotes compared with the dRsf-13602 heterozygotes.
(6.60 MB TIF)

Figure S4 No significant difference was detectable in the mRNA levels of Domino, H2Av and dReptin between wild type (WT) and the dRsf-1 mutant flies.
The expression levels of these three genes were measured by semi-quantitative RT-PCR. The level of transcripts from each gene was normalized by using the value of an internal standard Act5C. The levels of Domino, H2Av and dReptin transcripts were not significantly reduced in the dRsf-1 mutant embryos compared with WT.
(3.20 MB TIF)

Table S1 List of oligonucleotides used in this study.
(0.04 MB XLS)

Text S1 Supplementary materials and methods
(0.03 MB DOC)
