# Supplementary figures and images for: RSF Governs Silent Chromatin Formation via Histone H2Av Replacement
Source: PLoS Genet. 2008 Feb 29;4(2):e1000011. doi: 10.1371/journal.pgen.1000011 (PMC2265536; doi:10.1371/journal.pgen.1000011)

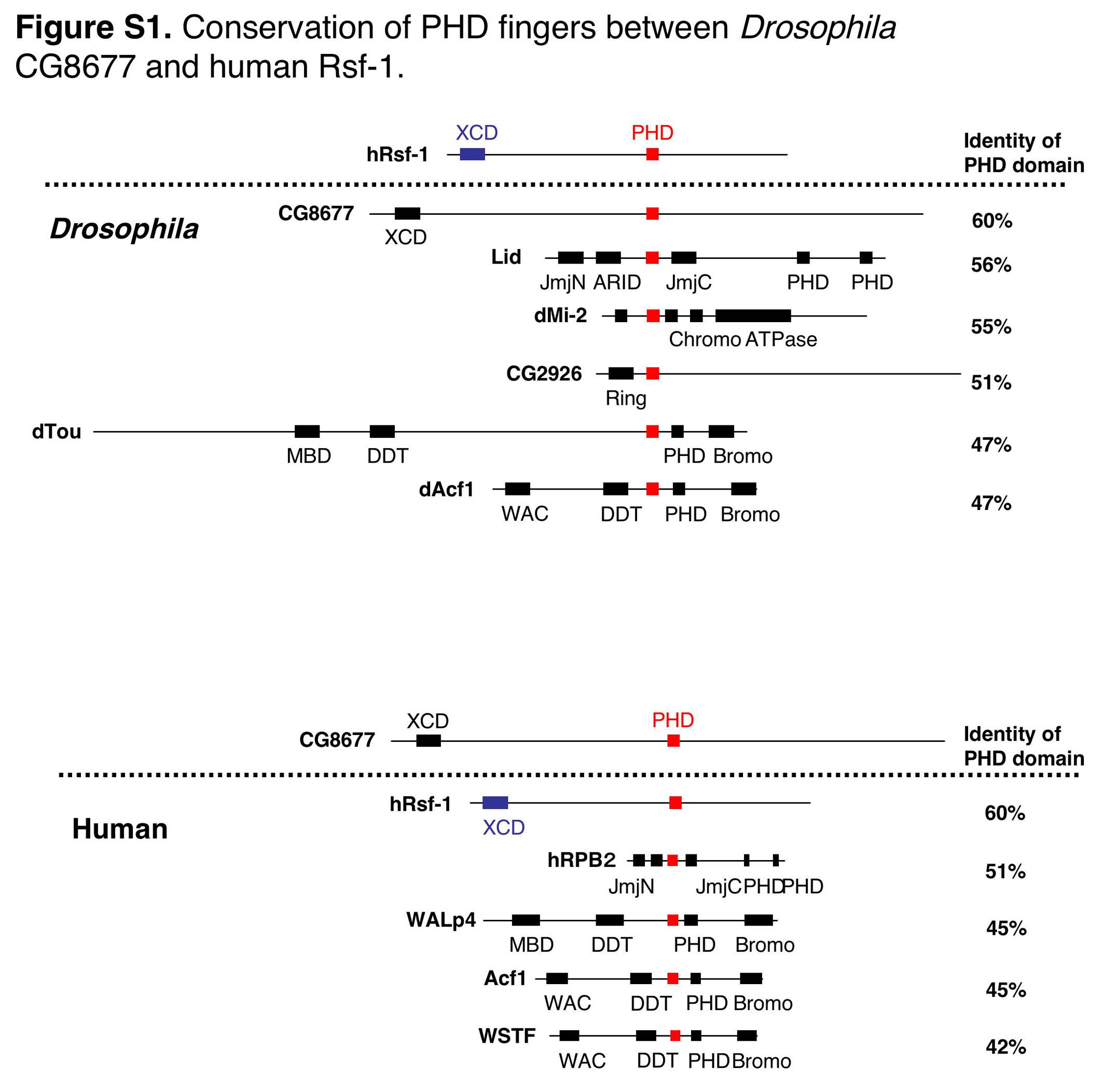

Supplement: Figure S1 — Conservation of PHD fingers between Drosophila CG8677 and human Rsf-1. Amino acid identities in PHD fingers were compared between various Drosophila and human proteins. Upper panel: Among Drosophila proteins, the PHD finger in CG8677 exhibits the highest homology with that in hRsf-1. Lower panel: Among human proteins, the domain in hRsf-1 shows the highest homology with that in CG8677. (2.45 MB TIF) [file pgen.1000011.s001.tif]

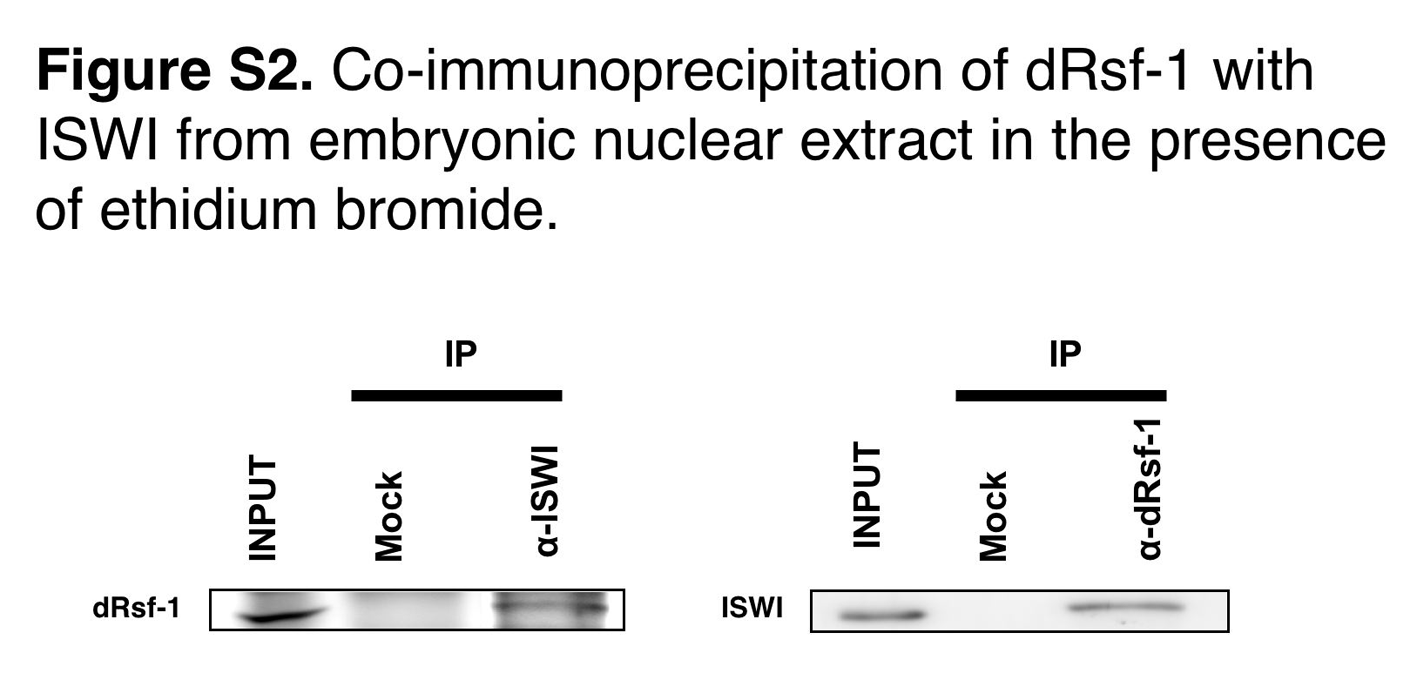

Supplement: Figure S2 — Co-immunoprecipitation of dRsf-1 with ISWI from embryonic nuclear extract in the presence of ethidium bromide (EtBr). Immunoprecipitation was carried out with anti-dRsf-1 antibodies, anti-ISWI antibodies or control IgG; subsequently western blotting of the immunoprecipitates was probed with anti-ISWI antibodies or anti-dRsf-1 antibodies. To prevent of nucleic acids-dependent protein interactions, the extract was incubated with 80 µg/ml EtBr on ice for 1 hr before the immunoprecipitation. Input represents 10% of the starting extract used for the immunoprecipitation. (0.79 MB TIF) [file pgen.1000011.s002.tif]

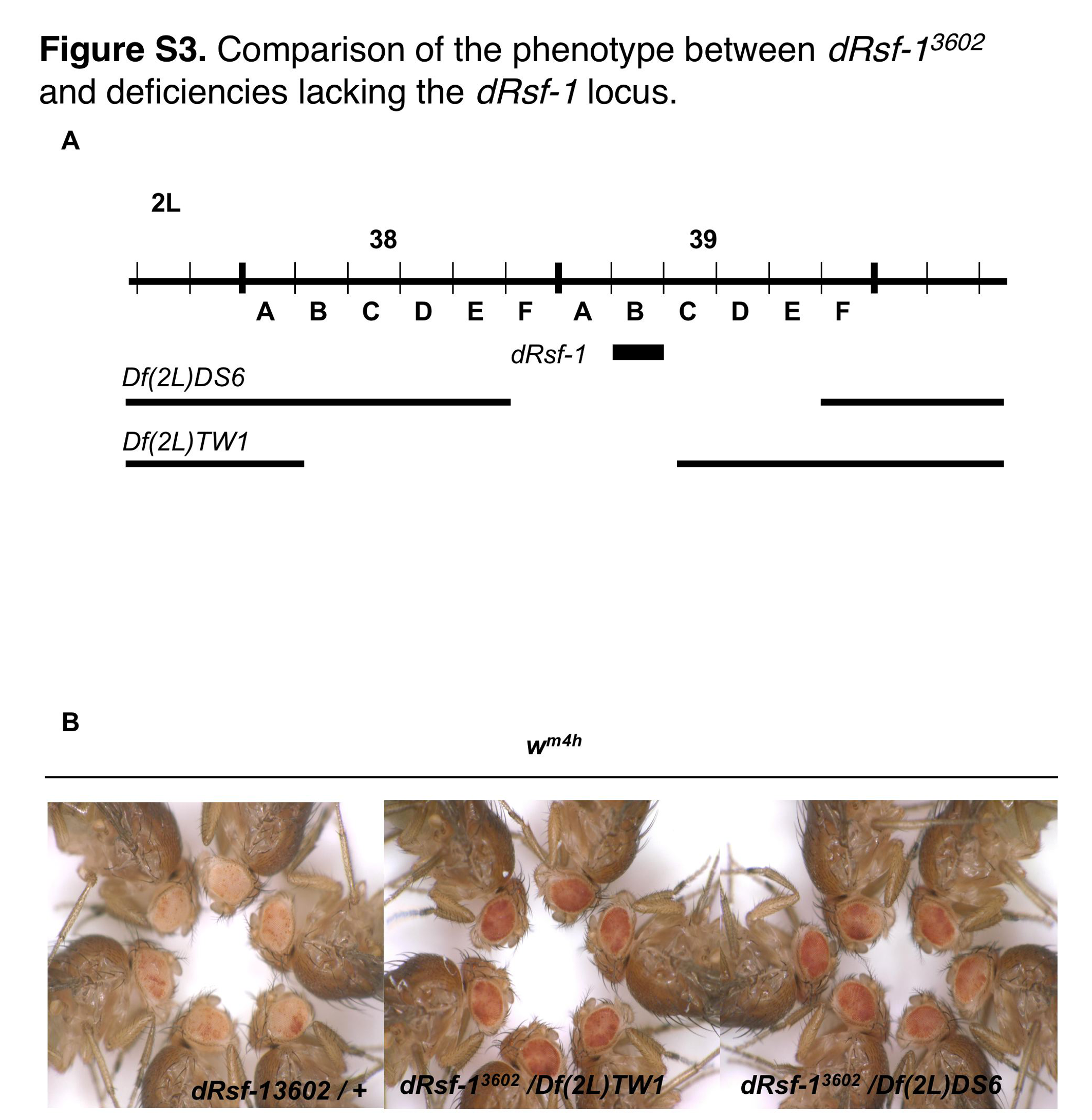

Supplement: Figure S3 — Comparison of the phenotype between dRsf-13602 and deficiencies lacking the dRsf-1 locus. (A) Schematic representation of the cytological region 38-39. Df(2L)DS6 deletes the 38E2-39E7 region and Df(2L)TW1 deletes the 38A7-39C3 region. (B) The double heterozygotes of dRsf-13602 and deficiency Df(2L)DS6 or Df(2L)TW1 suppressed the variegation strongly in a similar manner as the dRsf-13602 homozygotes compared with the dRsf-13602 heterozygotes. (6.60 MB TIF) [file pgen.1000011.s003.tif]

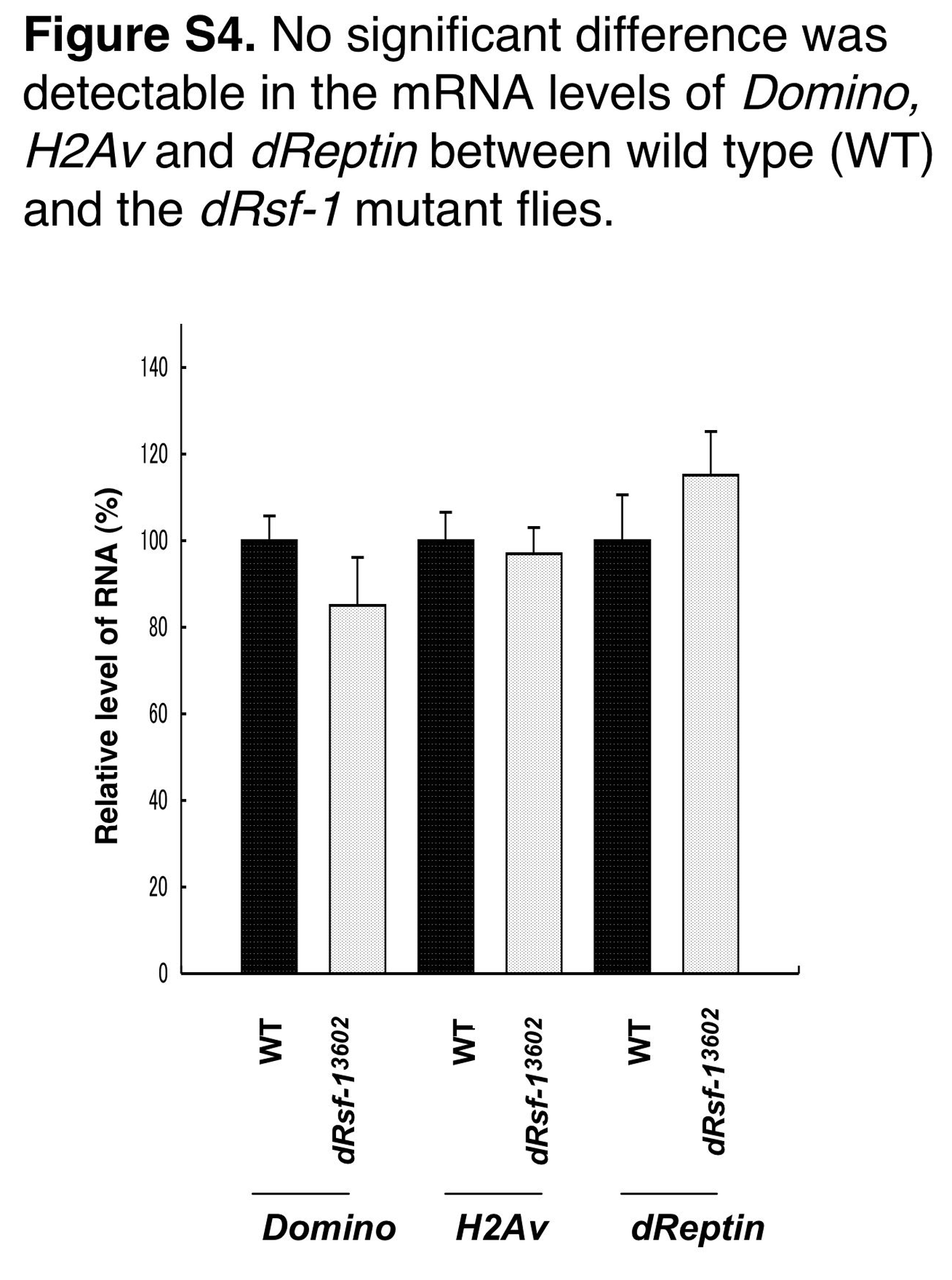

Supplement: Figure S4 — No significant difference was detectable in the mRNA levels of Domino, H2Av and dReptin between wild type (WT) and the dRsf-1 mutant flies. The expression levels of these three genes were measured by semi-quantitative RT-PCR. The level of transcripts from each gene was normalized by using the value of an internal standard Act5C. The levels of Domino, H2Av and dReptin transcripts were not significantly reduced in the dRsf-1 mutant embryos compared with WT. (3.20 MB TIF) [file pgen.1000011.s004.tif]
