# Supplementary material for: RSF Governs Silent Chromatin Formation via Histone H2Av Replacement
Source: PLoS Genet. 2008 Feb 29;4(2):e1000011. doi: 10.1371/journal.pgen.1000011 (PMC2265536; doi:10.1371/journal.pgen.1000011)
Supplement: Text S1 — Supplementary materials and methods (0.03 MB DOC) [file pgen.1000011.s006.doc]

**Supplementary Materials and Methods**

**Genetic analysis**

*Df(2L)TW1/Cyo* and *Df(2L)DS6/SM6a* were obtained from DGRC. All crosses were performed at 25℃. Male eyes were observed at 24 hours after eclosion.

**Accession numbers**

The Flybase ([http://flybase.bio.indiana.edu](http://flybase.bio.indiana.edu/)) accession numbers of the genes and gene products discussed in this paper are: *Actin 5C*, FBgn0000042; *dMRG15*, FBgn0027378; *Domino*, FBgn0020306; *dReptin*, FBgn0040075; *Drosophila Rsf-1* (*dRsf-1*, *CG8677*), FBgn0026577; *dTip60*, FBgn0026080; *Enhancer of Polycomb (E(Pc)),* FBgn0000581; *Histone H2A*, FBgn0001196; *Histone H2A variant* (*H2Av*), FBgn0001197; *Histone H3*, FBgn0001199; *ISWI*, FBgn0011604; *white*, FBgn0003996.
